# Helicobacter pylori as a Cause of Iron Deficiency in a Menstruating Woman in a Primary Care Setting

**DOI:** 10.7759/cureus.107354

**Published:** 2026-04-19

**Authors:** Nikhil A Nandkumar, Aruna Chakrala

**Affiliations:** 1 Neuroscience, University College London, London, GBR; 2 Internal Medicine, Plainsboro Princeton Medical Associates, Plainsboro, USA

**Keywords:** achlorhydria, atrophic gastritis, helicobacter pylori, iron deficiency anemia, thrombocytopenia

## Abstract

*Helicobacter pylori (H. pylori)* bacterial infections are moderately common clinical entities. They infect the mucosa lining of the antrum and cause pain and inflammation. The exact transmission of *H. pylori* remains unclear, though fecal-oral and oral-oral routes via contaminated food or water are considered common. Research over the past three decades has linked *H. pylori* infection to gastric cancer, micronutrient malabsorption, and malnutrition. Iron deficiency anemia is also often linked to *H. pylori* infection, where absorption is inadequate, or losses exceed absorption, eventually depleting iron stores and leading to anemia. Although the literature has widely studied this condition, diagnosing it upon clinical presentation may be challenging due to the various factors, causes, and tests required for an accurate diagnosis. We present the case of a 43-year-old female who initially presented to the clinic with nausea and fatigue, which turned out to be iron deficiency caused by *H. pylori*. An *H. pylori* breath test confirmed the infection and led to the prescription of a proton-pump inhibitor and a two-week course of an antibiotic called Prevpac. A repeat breath test one month later showed effective eradication of the bacterial infection and improvement in serum iron levels. This report highlights the diagnostic process and clinical considerations involved in identifying and managing the *H. pylori* infection.

## Introduction

*Helicobacter pylori (H. pylori)* is a gram-negative, helical, rod or curved-shaped, flagellated bacterium that can penetrate the mucus lining of the stomach and infect it [1]. Many patients are asymptomatic, and studies have shown that this bacterial infection can be transmitted via human contact with saliva, stool, or vomit [2]. It is correlated with living conditions such as crowdedness, lack of clean water supply, and cohabiting with an individual from a lower socioeconomic background in a developing country [3]. Such infections can cause atrophic gastritis, ulcers, or even gastric cancers such as mucosa-associated lymphoid tissue (MALT) [4]. A few studies have shown an association of developing anemia from an *H. pylori* infection, usually due to vitamin B_12_ or iron deficiency [5,6]. The global prevalence of *H. pylori *infection is 43.9% in adults and 35.1% in children and adolescents [7]. About 2% of these infected individuals go on to develop gastric cancers [3]. 

Beyond its well-known gastrointestinal manifestations, *H. pylori* infection can impair nutrient absorption, particularly iron, leading to systemic conditions, such as iron deficiency anemia (IDA), even in patients without prominent gastrointestinal symptoms. IDA is also common in menstruating women and is often attributed to menstrual blood loss [8]. However, it is poorly understood how unexplained gastrointestinal symptoms or nutritional deficiencies can arise in otherwise healthy individuals. This case emphasizes the need for a broader differential diagnosis in patients with refractory or unexplained iron deficiency. Notably, this case demonstrates that a noninvasive urea breath test can be used as an effective initial diagnostic tool in patients with unexplained iron deficiency or vague gastric symptoms, allowing for the identification and treatment of *H. pylori* without immediate reliance on upper endoscopy (EGD) or colonoscopy. In patients without gastrointestinal bleeding or unintentional weight loss, this approach may improve the efficacy of evaluation while reducing the patient burden. By illustrating this diagnosis, this case highlights a clinically relevant and non-invasive regimen, potentially identifying a reversible cause of anemia that may otherwise be missed in routine evaluation.

## Case presentation

We present the case of a 43-year-old Indian female who is a vegetarian with a 4-year history of chronic IDA. Within those four years, the patient was overlooked for more than three years due to a lack of urgent symptomatic effects. However, approximately six months prior to diagnosis, the patient developed intermittent cramping and bloating, accompanied by poor nutritional intake and dehydration. She also reported moderate constipation and progressive fatigue. Over time, she noted associated symptoms, including nausea, lightheadedness, and dizziness, which worsened about three months before diagnosis. Hair loss was also observed during this period, raising concern for underlying nutritional deficiency, particularly IDA. To address nutritional deficiency issues, she was advised to take over-the-counter B-vitamin supplementation, increase her intake of eggs and dairy, and receive intramuscular vitamin B_12_ injections. For constipation, she was instructed to use Miralax, with Senna 8.6 mg prescribed if symptoms persisted.

A week before diagnosis, she presented to the clinic for a follow-up evaluation of her laboratory results. Her chief complaints at the time included persistent nausea, fatigue, and dizziness. Intermittent cramping and abdominal pain were also noted at this time. On examination, her vital signs were within normal limits, with a weight of 134.4 lbs and a blood pressure of 118/78 mm Hg. Physical examination revealed mild right upper- and left lower-quadrant abdominal tenderness without rebound or guarding. Mucus membranes were noted to be dry, consistent with dehydration. Notably, classic signs of iron deficiency, such as pallor, pica, and brittle nails, were absent. Cardiovascular examination, including an ECG, was unremarkable, with no evidence of tachycardia or bradycardia. No reported signs of gastrointestinal bleeding were indicated. Although she has had a medical history of hypothyroidism, fatigue beyond baseline, weight gain, myalgias, skin changes, or goiter were not observed.

Laboratory investigations, starting around the time of her symptoms, revealed a markedly low serum ferritin level at 5 ng/mL, decreased total serum iron at 24 mcg/dL, elevated total iron binding capacity (TIBC) at 433 mcg/dL, and low percent saturation at 6%. This was also compounded by low hemoglobin at 9.7 g/dL and hematocrit at 33%, further indicating IDA. However, vitamin B_12_ deficiency was not detected, with a value of 698 pg/mL, and platelet counts were normal at 295 per mL of blood, indicating her reported symptoms could not be attributed to thrombocytopenia and could be attributed to IDA or other causes. A notable finding was an elevated fasting gastrin level at 146 pg/mL, which, in conjunction with her abdominal symptoms, raised suspicion for gastritis. As a result, further evaluation with a gastroenterologist was advised to rule out autoimmune metaplastic atrophic gastritis (AMAG). Before the gastroenterology consult, the patient was advised to begin iron supplementation with ferrous sulfate 325 mg to maintain ferritin levels. Further evaluation by the gastroenterologist ruled out celiac disease and AMAG. Subsequent diagnostic workup included a urea breath test, which returned positive for *H. pylori* infection. This finding provided a plausible etiology for her chronic gastritis and associated micronutrient deficiencies. The patient was prescribed a 14-day course of Prevpac, consisting of amoxicillin, clarithromycin, and a proton pump inhibitor (lansoprazole), to eradicate the infection and normalize her elevated gastrin levels. A follow-up urea breath test was scheduled one month after completion of therapy to confirm successful eradication of *H. pylori*. The patient was counseled on adherence to medication and advised dietary modifications to support gastrointestinal recovery. After complete eradication of her gastrointestinal symptoms three months later, only ferritin, iron % saturation, and vitamin B_12 _were outside the reference range, with values at 10 ng/mL, 11%, and 992 pg/mL, respectively. An interesting finding was the improvement of anemia to 11.9 g/dL hemoglobin and 36.6% hematocrit, possibly signifying the importance of early *H. pylori* testing. Table 1 summarizes her initial laboratory findings.

**Table 1:** Laboratory findings Blood levels for CBC, gastrin, iron panel, and B_12 _were recorded before and after treatment. Treatment was administered after observing initial values and lasted one month. Repeat blood work was administered every three months after treatment ended. The reference range is noted for comparison of test values and normal values. The CBC, gastrin, and ferritin test values significantly improved over the course of the treatment and are expected to improve over the next four to eight months. Values in bold represent those outside the reference normal range. Values that are not in bold indicate a value within the normal reference range. (L) = Below normal range; (H) = Above normal range

Blood Test	Before Treatment	After Treatment	Reference Range
Gastrin	146 pg/mL (H)	38 pg/mL	<=100 pg/mL
Ferritin	5 ng/mL (L)	10 ng/mL (L)	Males: 24-336 ng/mL Females: 24-307 ng/mL
Total Iron	24 mcg/dL (L)	43 mcg/dL	Males: 50-150 mcg/dL Females: 35-145 mcg/dL
Iron Binding Capacity	433 mcg/dL (H)	390 mcg/dL	Males: 240-450 mcg/dL Females: 215-400 mcg/dL
% saturation	6% (L)	11% (L)	Males: 20-50% Women: 15-45%
Serum B_12_	698 pg/mL	992 pg/mL (H)	160-950 (pg/mL)
Hemoglobin	9.7 g/dL (L)	11.9 g/dL	11.7 - 15.5 (g/dL)
Hematocrit	33.00% (L)	36.60%	35.9%-46%
Platelet Count	295/mL blood	249/mL blood	140-400 (/mL blood)

Although gastritis, anemia, and *H. pylori* infection were successfully treated, low ferritin levels persisted until the following year. She initiated IV iron dextran therapy after the completion of Prevpac, but only saw a slight benefit in laboratory work. The patient reported irregular menstrual cycles, including delays of one to two weeks, beginning three years ago. Gynecologic evaluation, including abdominal ultrasound, identified a single uterine fibroid. Subsequently, two years later, she reported heavy menstrual bleeding with irregular cycles. While iron levels showed slight improvement, heavy menstrual bleeding was considered a major contributor to persistent low ferritin. Corvita 150 mg daily was prescribed for three months to monitor improvement, and consultation with a gynecologist was recommended. She was also advised to take two over-the-counter iron supplements during each menstrual cycle.

Following treatment with oral iron supplementation, iron dextran injection, and Prevpac therapy, ferritin levels improved modestly, anemia improved slightly into the normal range, and *H. pylori* infection and gastritis were completely eradicated. Her symptoms, including fatigue, nausea, and bloating, also improved. No further treatment was required at that time. The post-treatment plan included continuation of Corvita to maintain ferritin levels while addressing irregular menstrual cycles and heavy menstrual bleeding with her gynecologist. This case demonstrates that *H. pylori* infection can underlie IDA in menstruating women without prominent initial gastrointestinal symptoms, and that urea breath tests may serve as an effective first-line diagnostic approach, potentially avoiding unnecessary endoscopic procedures while identifying a reversible cause of anemia.

## Discussion

Our patient was diagnosed with an *H. pylori* infection in a primary care clinic after trends in laboratory workup demonstrated IDA, which was followed by treatment of the underlying cause of the hematological deficiencies. The physician recommended a one-month follow-up and repeat lab work every three months to confirm treatment efficacy. Post-infection showed treatment efficacy through the complete eradication of gastrointestinal symptoms.

Before treatment, the patient exhibited classical features of IDA, including noticeably low ferritin (5 ng/mL), low serum iron (24 mcg/dL), elevated total iron-binding capacity (433 mcg/dL), and very low transferrin saturation (6%). This reflects depleted iron stores and an increase in iron-binding capacity, as the body compensates to maximize iron transport. The elevated gastrin level of 146 pg/mL supports the presence of gastritis, likely driven by *H. pylori*, which can impair gastric acid secretion and reduce iron absorption. Hemoglobin (9.7 g/dL) and hematocrit (33%) were also reduced, confirming established anemia. Normal platelet count and normal vitamin B_12_ at baseline help exclude alternative hematologic etiologies such as thrombocytopenia or isolated B_12_ deficiency. Following treatment, several key improvements were observed. Gastrin levels normalized from 146 pg/mL to 38 pg/mL, highlighting successful eradication of *H. pylori* and gastritis. Serum iron increased from 24 mcg/dL to 43 mcg/dL, TIBC decreased from 433 mcg/dL to 390 mcg/dL, and transferrin saturation improved from 6% to 11%, indicating partial improvement of iron availability and reduced compensatory iron-binding activity. Hemoglobin also increased from 9.7 g/dL to 11.9 g/dL and hematocrit from 33% to 36.6%, reflecting effective recovery from anemia.

However, ferritin remained at low levels, highlighting that iron stores are replenished more slowly than circulating iron and hemoglobin. This lag is expected, particularly in the case of chronic depletion and ongoing losses such as menstrual bleeding. The persistently low ferritin despite treatment suggests that, although absorption has improved following *H. pylori* eradication, total body iron stores have not yet been fully replenished. The elevated vitamin B_12_ can be due to the intramuscular B_12_ injections given due to her nutritional deficiencies. This pattern emphasizes the importance of addressing both the underlying cause and the need for continued iron supplementation during recovery.

However, many patients are asymptomatic, and routine testing is not recommended. Testing is indicated in dyspepsia, unexplained IDA, active peptic ulcer disease (PUD), history of PUD, symptoms of dyspepsia, and long-term nonsteroidal anti-inflammatory drugs (NSAID) use, among others. IDA has increasingly been associated with *H. pylori* infections and has often been known to be overlooked [6,9]. Recently, a meta-analysis of several case-control studies has been done to investigate this relationship. One of these studies showed that patients have an increased risk of IDA when an *H. pylori* infection persists [10]. Similarly, eradication of *H. pylori* infection showed recovery from IDA [5,8]. During the eradication therapy for IDA, results have shown significant differences between the baseline and endpoint of serum iron, serum ferritin, and hemoglobin, indicating therapeutic benefits for anemia. Several mechanisms explain how *H. pylori* infections cause IDA. First, PUD, hemorrhagic gastritis, and gastric adenocarcinoma can cause increased iron loss [11]. Also, it has been shown that *H. pylori*-related gastritis may decrease hydrochloric acid secretion due to stomach atrophy, which causes reduced absorption of iron from food intake [12]. Multiple studies also show that *H. pylori* causes a decrease in the concentration of ascorbic acid, a known enhancer of serum iron absorption [13,14]. These studies have shown significance in studying this relationship further through both mechanistic and clinical approaches, which can be applied to advancing treatment methods. Lastly, the CagA protein of *H. pylori* has been shown to acquire increasing amounts of iron from interstitial holotransferrin [15]. The association of *H. pylori* causing IDA has grown conclusive in many studies. Current guidelines now recommend eradication of H. pylori in patients presenting with unexplained IDA [16,17].

Achlorhydria and hypochlorhydria have also been associated with vitamin B_12_ deficiency due to anemia caused by *H. pylori* [18]. The presence of *H. pylori* infection of gastric epithelial cells represses K^+^-ATPase alpha subunit gene expression, leading to transient hypochlorhydria [19]. One study showed that 67.4% of patients with an *H. pylori* infection also had vitamin B_12_ deficiencies [20]. Another study showed that patients with low serum B_12_ levels had a higher prevalence of *H. pylori* infection [21]. Although many studies show the association between serum B_12_ levels and *H. pylori*, there have not been many studies showing significant effects of *H. pylori* eradication on improving serum B_12_ levels.

IDA can also be explained by excessive blood loss during menstruation. When exploring heavy menstrual bleeding and anemia, a recent review shows that hormonal contraceptives have significantly reduced the blood loss as a result of heavy menstrual bleeding and anemia, indicating a possible avenue for the patient [22]. In our patient, anemia could have been quickly and easily attributed to her irregular periods and heavy menses. However, careful investigation of her other gastrointestinal symptoms prompted further evaluation, allowing the proper diagnosis of H. pylori. The combination of studies linking menstruation to IDA and *H. pylori *to IDA demonstrates the importance of recognizing that anemia and gastrointestinal symptoms may coexist. Current guidelines emphasize that IDA warrants evaluation for underlying causes beyond apparent risk factors, such as menstrual blood loss, including *H. pylori* [23], and medical professionals should not stop evaluation at menstrual history alone when anemia is refractory or unexplained.

Accurately diagnosing an infection concludes with the urea breath test. This test uses a pill containing tagged carbon molecules, which are released if *H. pylori* is present. The patient blows into a bag, and the body absorbs the carbon released when breathing out [24]. Various other methods have been found to effectively diagnose *H. pylori* infection, including endoscopy followed by immediate histological and serological results analysis [25].

The advancement in testing and treatment methods has decreased the prevalence over the past few years. Antibiotic treatments and proton pump inhibitors, which inhibit gastric acid production, have been shown to be a significant reason for it [26]. A combination antibiotic treatment uses amoxicillin, lansoprazole, and clarithromycin to eradicate the bacterial infection. Clarithromycin interacts with the peptidyl transferase loop in the V domain of the 23S ribosomal RNA, thus inhibiting bacterial protein synthesis [27]. Amoxicillin interacts with penicillin-binding proteins (PBP), inhibiting the synthesis of the cell wall and leading to bacterial disintegration [27]. Using this drug has been proven effective since *H. pylori* has been known to contain multiple PBPs. Lansoprazole, a frequently administered proton pump inhibitor (PPI), has sufficiently suppressed acid production [26]. The literature provides evidence for using PPIs to inhibit gastric acid secretion in eradicating *H. pylori*. The coccoid form of *H. pylori* develops in an acidic environment (pH 3-6) and is resistant to antibiotics [26]. Therefore, it is vital to increase the intragastric pH. Vonoprazan, a type of PPI, acts as a competitive inhibitor of H^+^/K^+^-ATPase, present on the luminal membrane of gastric epithelial cells, and demonstrates high efficacy in inhibiting acid production [26]. However, studies have not proven generalizable across all regions worldwide, and more studies should also be done in Western countries. The rapid and efficient diagnosis of IDA is a crucial indicator for identifying its causes, such as *H. pylori*, and various treatment regimens should not be underestimated.

## Conclusions

*H. pylori* infection can cause iron deficiency through several mechanisms, including chronic gastric inflammation, hypochlorhydria, and direct bacterial consumption of iron, impairing absorption. Importantly, IDA may be the only clinical manifestation of infection, even without typical gastrointestinal symptoms. Beyond menstruating women, this also highlights the need to consider *H. pylori* testing in patients with unexplained iron deficiency, particularly in non-menstruating adults, men, and postmenopausal women. Eradication of the infection often leads to improvement in iron stores without prolonged supplementation, suggesting it as a reversible cause of anemia. Routine *H. pylori* screening could be effective in iron deficiency workups, particularly in high-prevalence areas, and broader eradication efforts may have public health benefits beyond reducing gastric cancer risk. This case demonstrates how deficiencies in serum iron and hemoglobin, along with elevated gastrin, prompted targeted testing, ultimately leading to the diagnosis and successful treatment of *H. pylori* infection. Further studies are required to examine why patients don’t experience direct symptoms of *H. pylori* infection. However, this case highlights the importance of broadening the differential diagnosis of IDA in menstruating women to include overlooked infectious etiologies, such as *H. pylori*, emphasizing that timely recognition and treatment of these reversible causes can significantly improve patient outcomes.
